# Catheter-based distal sciatic nerve block in patients with Charcot-Marie-Tooth disease

**DOI:** 10.1186/1471-2253-14-8

**Published:** 2014-02-14

**Authors:** Hubert J Schmitt, Sandra Huberth, Horst Huber, Tino Münster

**Affiliations:** 1 Department of Anesthesia, Friedrich-Alexander Universität, Krankenhausstraße 12, D-91054 Erlangen, Germany; 2 Department of Anesthesia, Waldkrankenhaus St. Marien, Rathsberger Str. 57, D-91054 Erlangen, Germany

**Keywords:** Charcot-Marie-Tooth disease, Peripheral neuropathies, Peripheral nerve block

## Abstract

**Background:**

The use of peripheral nerve blocks in patients with Charcot-Marie-Tooth (CMT) disease is scarcely reported; however, when performed it has proven to be effective for postoperative pain control.

**Methods:**

A distal catheter-based sciatic nerve block for postoperative pain control was offered to 27 consecutive CMT patients scheduled for elective foot surgery. 18 of the 27 CMT patients consented to the offered sciatic nerve block. Localization of the sciatic nerve was guided by a nerve stimulator. The threshold current required to generate a motor response was assessed and a catheter inserted. Postoperative pain was assessed by recording the dose of analgesics to maintain visual analog score < 3 the next 48 hours. On demand patients received boluses of ropivacaine (2 mg/mL) via the catheter and/or analgesics in case of insufficient pain relief. Total postoperative ropivacaine dosage and analgesic consumption were recorded. About one year after the block patients were contacted to report their actual status by self-assessment.

**Results:**

In 17 patients a catheter could be placed. In 7 patients placement of the catheter was difficult (several attempts, high electrical impedance). Patients with nerve block had lower analgesics consumption compared to patients without a block. Surprisingly, the 7 patients with “difficult” catheter-placement had the overall lowest ropivacaine and analgesics consumption compared to all other patients with or without peripheral block. No anesthesia related complications were reported by the questionnaire.

**Conclusions:**

In our small series catheter-based distal sciatic block within CMT patients had safely been used for pain relief up to three days. The infusion of local anesthetics via a catheter was not associated with any complication.

## Background

Charcot-Marie-Tooth (CMT) disease is a heterogeneous group of inherited peripheral neuropathies with an estimated prevalence of 1 in 2500 people. The disease is caused by duplication on chromosome 17p11.2 [1]. Demyelination of peripheral nerves cause marked slowing of nerve conduction velocity and a decrease in compound motor and sensory nerve action potentials. Patients suffer from slowly progressive distal muscle wasting, weakness and sensory loss, first affecting the feet and legs [2]. Foot deformities cause gait disturbances and balance problems. To overcome these problems CMT patients often have to undergo foot surgery to improve their walking ability.

Peripheral nerve blocks provide good postoperative analgesia and support postoperative rehabilitation with a high rate of patient satisfaction. Many anesthesiologists fear permanent neurological injury or aggravation of the underlying disease when performing regional anesthesia in patients with pre-existing neuropathy [3,4]. In contrast, as far as the authors know, there is no case published documenting any adverse outcome following peripheral nerve block in CMT patients. We therefore prospectively studied the efficacy, safety and patient satisfaction of distal sciatic block in CMT patients scheduled for foot surgery. In this article we report on our experience in performing a distal sciatic block in a small series of CMT patients.

## Methods

In our hospital all patients scheduled for foot surgery are offered sciatic nerve block for acute postoperative pain control. Patients with CMT are not excluded in principle from this procedure. Between January 2003 and November 2010, 27 consecutive patients, diagnosed with CMT and scheduled to receive anesthesia for elective foot surgery were considered eligible for sciatic nerve block for postoperative analgesia. The catheter-based nerve block was offered to all 27 patients followed by detailed discussion regarding procedure, potential benefits and possible risks. 18 of the 27 CMT patients consented to the offered sciatic nerve block. The study was approved by the ethics committee of the Friedrich-Alexander University Erlangen-Nuremberg and written informed consent was obtained from all patients. For ethical reasons randomization of the participants was not possible.

### Anesthetic management

In the operation theatre all patients were monitored throughout the procedure by electrocardiography, non-invasive blood pressure, and pulse oximetry. After an intravenous access had been established patients who had consented to neural block were placed in the lateral position with the leg to be blocked at the top and extended at the knee joint. To block the distal sciatic nerve the lateral popliteal fossa approach was used. All blocks were performed by two anesthesiologists with substantial expertise in regional anesthesia. After skin preparation, a 18G insulated needle (Contiplex D system, Braun Melsungen, Germany) was inserted about 8-10 cm cephalad to the lateral femoral epicondyle in the groove between the biceps femoris and the vastus lateralis muscles. The needle was directed about 45° cephalad until a dorsal or plantar flexion of the foot was obtained. The initial stimulating current was set to 1.0 mA with impulse duration of 1.0 ms and a frequency of 2 Hz. After establishing initial muscle contractions the needle was carefully repositioned in order to obtain the minimum current strength and duration (threshold) - with first reducing stimulus duration before current strength - necessary to generate visible muscle contractions. This threshold just enough to generate a twitch was recorded and the product of current and duration was calculated. The settings of the nerve stimulator, number of attempts, technical problems and complications were documented. Stimulus duration of 0.3 ms was applied as cut-off value. If stimulus duration was higher the block was defined as “high threshold” block. Corresponding, in “low threshold”-blocks the stimulus duration could be reduced below 0.3 ms.

The catheter (20G, Contiplex D system, Braun Melsungen, Germany) was then inserted through the needle and advanced about 4-5 cm over the tip of the needle. Following negative aspiration a test dose consisting of 5 ml of ropivacaine (3.75 mg/mL) was administered through the catheter. Given a negative test dose, a total dose of 20 ml of ropivacaine (3.75 mg/mL) was given in aliquots through the catheter.

General anesthesia was induced and maintained as total intravenous anesthesia with propofol and remifentanil titrated to effect except in two patients. These two patients (both with peripheral block) specifically requested spinal anesthesia. All patients without a peripheral block wished general anesthesia. At the end of surgery and complete emergence from anesthesia patients were transferred to the post-anesthesia care unit.

### Postoperative management

For the next 48 hours patient’s postoperative pain was measured by the means of a numerical 11-point scale (NRS) ranging from zero (no pain) to 10 (worst pain). During this time patients provided with a sciatic nerve catheter received on demand and up to 4 boluses of 20 ml of ropivacaine (2 mg/mL) into the catheter. Prior the bolus was given by an anesthesiologist patients were asked whether motor function and sensitivity of the leg were as “normal”. Patients without a regional block or with insufficient pain relief (NRS >3) by the regional block received supplementary pain therapy by intravenous paracetamol and piritramide. Total postoperative ropivacaine dosage and total analgesic drug consumption of every patient were recorded.

To identify any changes regarding their disorder all patients were contacted by phone about one year after surgery. The patients were asked with the help of a standardized basic questionnaire to assess their actual status by self-assessment. The questions covered details like ability to walk, and necessity of additional assist devices. Patients were asked to classify the actual with the pre-operative status as: improvement, no change, or worsening. Patients with a peripheral block were asked if they would prefer regional block again.

Postoperative demand for analgesic drugs was compared between patients with or without peripheral block using Mann–Whitney *U* test. Statistical analysis was performed using the STATA statistical package (STATA Corporation, College Station, Tex). A p-value <0.05 was considered significant.

## Results

The demographic variables, including age, gender, weight, height, time since CMT had been diagnosed, and ASA classification are summarized in Table 1. There was no significant difference regarding demographic variables between patients with or without a peripheral block. According to the cut-off value of 0.3 ms for stimulus duration patients with peripheral block were divided into two groups with high and low threshold. 7 out of the 17 patients have been allocated to the high-threshold group including one patient were the catheter could not be placed due to insufficient twitch elicitation. Table 2 depicts electrophysiological data for peripheral nerve location. Comparing both groups with nerve blocks, patients with high threshold blocks requested significant lower amounts of ropivacaine, while there were no differences regarding intravenous paracetamol and piritramide administration. Comparing the amount of intravenous analgesics between patients with and without nerve blocks, only patients with high threshold blocks showed significant less demand of piritramide. Table 2 shows all data regarding postoperative pain management. 20 of the 27 patients responded to the questionnaire one year after surgery. Details of the questionnaire are summarized in Table 3. No anesthesia related complications were reported in the questionnaire by the patients.

**Table 1:** Demographic data of the patients

**Group**	**Age (y)**	**Height (cm)**	**Weight (kg)**	**Gender (F/M)**	**Time since diagnosis (month)**	**ASA classification I/II/III**	**CMT type 1/2/unknown**	**Osteotomies/soft tissue surgery**
**Patients with block n = 17**	39 (18-59)	170 (160-183)	68 (50-86)	7/10	120 (8-351)	4/13/0	14/1/2	15/2
**Patients without block n = 10**	20 (18-51)	173 (158-190)	69 (43-103)	5/5	148 (17-528)	2/7/1	7/1/2	9/1

The values are given as median (range) or number.

**Table 2:** Electrophysiological data and perioperative analgesics requirement

**Group**	**Current (mA)**	**Stimulus duration (ms)**	**Charge (mAxms)**	**Ropivacaine (mg)**	**Piritramide (mg/kg)**	**Paracetamol (mg/kg)**
**Patients with low threshold block n = 10**	0.3 (0.2–0.7)	0.1(0.1)	0.03 (0,02-0.07)	370 (120-720)	0.07 (0-0.28)	24 (0-59)
**Patients with high threshold block n = 7**	1* (0.3-5)	1.0* (0.3-1.0)	0.6* (0.18-5)	100* (0-300)	0.05† (0-0.17)	0 (0-20)
**Patients without block n = 10**					0.2 (0.03-0.34)	18 (0-49)

The values are given as median (range). Analgesics are given as total amount of requirement over 48 hours postoperatively.

*p < 0.05 group ‘low’ *vs* ‘high’ threshold; Mann–Whitney *U* test.

†p < 0.05 group ‘high threshold *vs* no block; Mann–Whitney *U* test.

**Table 3:** Questionnaire

**Group**	**Block again Yes/no**	**Block associated complication (self-assessment)**	**Quality of life (self-assessment) better/no change/worse**	**No response to questionnaire**
**Patients with low threshold block n = 10**	7/1	none	6/2/0	2
**Patients with high threshold block n = 7**	1/5	none	3/3/0	1
**Patients without block n = 10**			5/2/0	3

The values are given as numbers.

## Discussion

In this study we investigated the efficacy, safety and patient satisfaction of a catheter-based distal sciatic block in CMT patients. The results of this study suggest that peripheral nerve block can safely be performed in patients suffering from CMT. Application of local anesthetics via a catheter up to three days provided sufficient analgesia without any complication.

Patients with CMT disease suffer from muscle wasting predominantly in the lower limbs. Hallmarks in electrophysiological findings are decreased motor nerve conduction velocity and overall reduced electrical excitability [5].

Even though there are no conclusive publications documenting the drawback of the use of regional anesthesia in CMT patients many anesthesiologists raise serious concerns over this theme. Regional anesthetic techniques in CMT disease have been anecdotally reported by case reports or small case series [6-10]. Remarkably, all these reports document significant pain relief by regional techniques, no side effects, and in many cases patient satisfaction (as far as reported). Our finding that patients without nerve block requested significant more analgesics than patients with nerve block was not surprisingly. However, the most striking finding of our study was the fact that patients with a high threshold (corresponding to high impedance) during block performance showed the overall lowest analgesic requirements. We speculate that different degree of demyelination of peripheral nerve fibers in our patients may be responsible for this phenomenon. In addition, there seems to be a high inter-individual variability of sensitivity to pain among CMT patients unrelated to the CMT type [11,12].

Electrical excitability and conduction velocity of peripheral nerves are intrinsically linked to an intact myelinated axon of peripheral nerves. The documented loss of integrity of the axon membrane in CMT [5] could explain the high threshold current in some patients performing a nerve block. In an experimentally study Tsui et al. [13] documented a significant increase in the threshold current to generate a motor response of a peripheral nerve if the needle was placed intraneurally. We speculate that a demyelinated nerve may in part mimic intraneural situation and so explain the different current thresholds reported in this study.

Most feared by anesthesiologists considering regional anesthesia in patients with pre-existing neurologic disorders is possible worsening of the underlying disease by a regional block. Reviewing the literature we found no report documenting such a case in CMT. To get a survey of the course of the disease in our patients we assessed patients using a standardized questionnaire about one year after anesthesia. Most of the patients reported an improvement of quality of life compared to their preoperative status. Some patients reported no change of their general status. Our questionnaire showed that acceptance of the peripheral block was closely associated with the ease of nerve localization. The majority of patients with easy localization (low threshold) would wish again a peripheral block; patient where several attempts with high thresholds were necessary will refuse a peripheral block in future.

The fact that no anesthesia related complication was reported encourages our view that if patients with CMT want regional block anesthesiologists should meet this demand. In so far our results confirm case reports and a case series where the uneventful use of central and regional anesthesia in CMT has been documented [6,7,10].

A limitation of this study is the fact that we have not used ultrasound for placement of the catheter, now widely accepted as gold standard. When designing and performing this study, ultrasound was not available at our institution. We are convinced that the use of ultrasound probably may have reduced the number of attempts and shorten the time for catheter placement.

## Conclusions

This study shows that in CMT patients a sciatic nerve block can safely be performed and may help to reduce postoperative opioid consumption. The infusion of local anesthetics up to three days via a catheter was not associated with any complication.

## Competing interests

The authors declare that they have no competing interests.

## Authors’ contributions

HJS- conceived the idea and designed the study, wrote the manuscript. SH- participated in the design of the study and assisted in data analysis, HH- made the coordination of the study, revised the manuscript. TM- designed the study, analyzed the data, and edited the manuscript. All authors read and approved the final manuscript.

## Pre-publication history

The pre-publication history for this paper can be accessed here:

http://www.biomedcentral.com/1471-2253/14/8/prepub
